# *Drosophila melanogaster dHCF* Interacts with both PcG and TrxG Epigenetic Regulators

**DOI:** 10.1371/journal.pone.0027479

**Published:** 2011-12-08

**Authors:** Sara Rodriguez-Jato, Ana Busturia, Winship Herr

**Affiliations:** 1 Center for Integrative Genomics, University of Lausanne, Génopode, Lausanne, Switzerland; 2 Centro de Biología Molecular "Severo Ochoa", Madrid, Spain; Centre National de la Recherche Scientifique, France

## Abstract

Repression and activation of gene transcription involves multiprotein complexes that modify chromatin structure. The integration of these complexes at regulatory sites can be assisted by co-factors that link them to DNA-bound transcriptional regulators. In humans, one such co-factor is the herpes simplex virus host-cell factor 1 (HCF-1), which is implicated in both activation and repression of transcription. We show here that disruption of the gene encoding the *Drosophila melanogaster* homolog of HCF-1, *dHCF*, leads to a pleiotropic phenotype involving lethality, sterility, small size, apoptosis, and morphological defects. In *Drosophila*, repressed and activated transcriptional states of cell fate-determining genes are maintained throughout development by Polycomb Group (PcG) and Trithorax Group (TrxG) genes, respectively. *dHCF* mutant flies display morphological phenotypes typical of TrxG mutants and *dHCF* interacts genetically with both PcG and TrxG genes. Thus, *dHCF* inactivation enhances the mutant phenotypes of the *Pc* PcG as well as *brm* and *mor* TrxG genes, suggesting that *dHCF* possesses Enhancer of TrxG and PcG (ETP) properties. Additionally, *dHCF* interacts with the previously established ETP gene *skd*. These pleiotropic phenotypes are consistent with broad roles for dHCF in both activation and repression of transcription during fly development.

## Introduction

Much of the early foundations of our understanding of genetic and epigenetic regulation of animal development originates from studies of the fruit fly *Drosophila melanogaster*. These studies revealed groups of genes with shared developmental functions. Two such well-known groups are the Polycomb group (PcG) and the Trithorax group (TrxG) whose members are generally involved in long-term maintenance of expression patterns of cell fate-determining genes, such as homeotic genes, during fly development (reviewed in [1]). PcG and TrxG proteins act primarily by controlling chromatin states through their incorporation into protein complexes possessing chromatin-modifying enzymatic activities. Consistent with the central role that PcG and TrxG proteins play during development, their function and corresponding protein complexes have been well conserved during evolution (reviewed in [2]).

In *Drosophila*, PcG-related protein complexes are associated with repression of gene transcription by mechanisms that include (i) direct modification of histones, (ii) recruitment of histone variants, and (iii) regulating ATP-dependent chromatin remodeling ([3] and reviewed in [4]). In contrast, TrxG-related protein complexes, while using similar mechanisms, generally support active gene transcription (reviewed in [2]). Genetically linking PcG and TrxG gene activities is a less well-characterized class of genes called Enhancer of Trithorax and Polycomb (ETP) that may act as co-factors of specific PcG and/or TrxG complexes in the activation and repression of subsets of cell-type and developmental stage-specific genes (reviewed in [1] and [5]). Here, we present a genetic analysis of the *Drosophila* homolog of the gene encoding the human herpes simplex virus (HSV) host-cell factor-1 (HCF-1) protein and show that it enhances phenotypes associated with PcG and TrxG mutants, thus displaying ETP properties.

Human HCF-1 is associated with the activation and repression of gene expression (reviewed in [6], [7], [8]). It possesses no known enzymatic nor DNA-binding activities, but rather is brought to specific promoters by association with DNA-sequence-specific transcription factors such as Sp1, GABP, YY1, Ronin/THAP11, and E2F1 and E2F4 [8], [9], [10], [11], [12], [13]. In turn, HCF-1 associates with and promotes the recruitment of chromatin-modifying activities such as Set1/Ash2 [14] and Mixed Lineage Leukemia (MLL)/Ash2 [15] Trx-related histone methyltransferases, MOF acetyltransferase [16] and Sin3A histone deacetylase [14]. HCF-1 appears to integrate DNA-sequence-specific transcription factors with specific combinations of chromatin modifying activities to both activate and repress transcription (see [8]).

Properties of HCF-1 have been highly conserved amongst animals. For example, the *Drosophila* homologue, dHCF, shares (i) a Kelch domain often responsible for transcription factor interaction, (ii) regions biased for basic (Basic) or acidic (Acidic) amino acids, (iii) fibronectin type 3 repeats, and (iv) a nuclear localization signal [17], [18]. In addition, although by different enzymes – O-GlcNAc transferase and taspase1, respectively [19], [20] – both HCF-1 and dHCF proteins undergo a process of proteolytic maturation to produce a heterodimeric complex of HCF_N_ and HCF_C_ subunits [17]. The conservation between human and *Drosophila* homologues goes beyond a structural similarity because both proteins have been shown to interact with common transcription factors [8], [17], and chromatin modifiers [14], [21]. This conservation between human and *Drosophila* HCF proteins as well as the rich genetic resources for studying epigenetic regulation afforded by the fly, led us to study the function of the *dHCF* gene in *Drosophila*.

## Results

**Figure 1:**
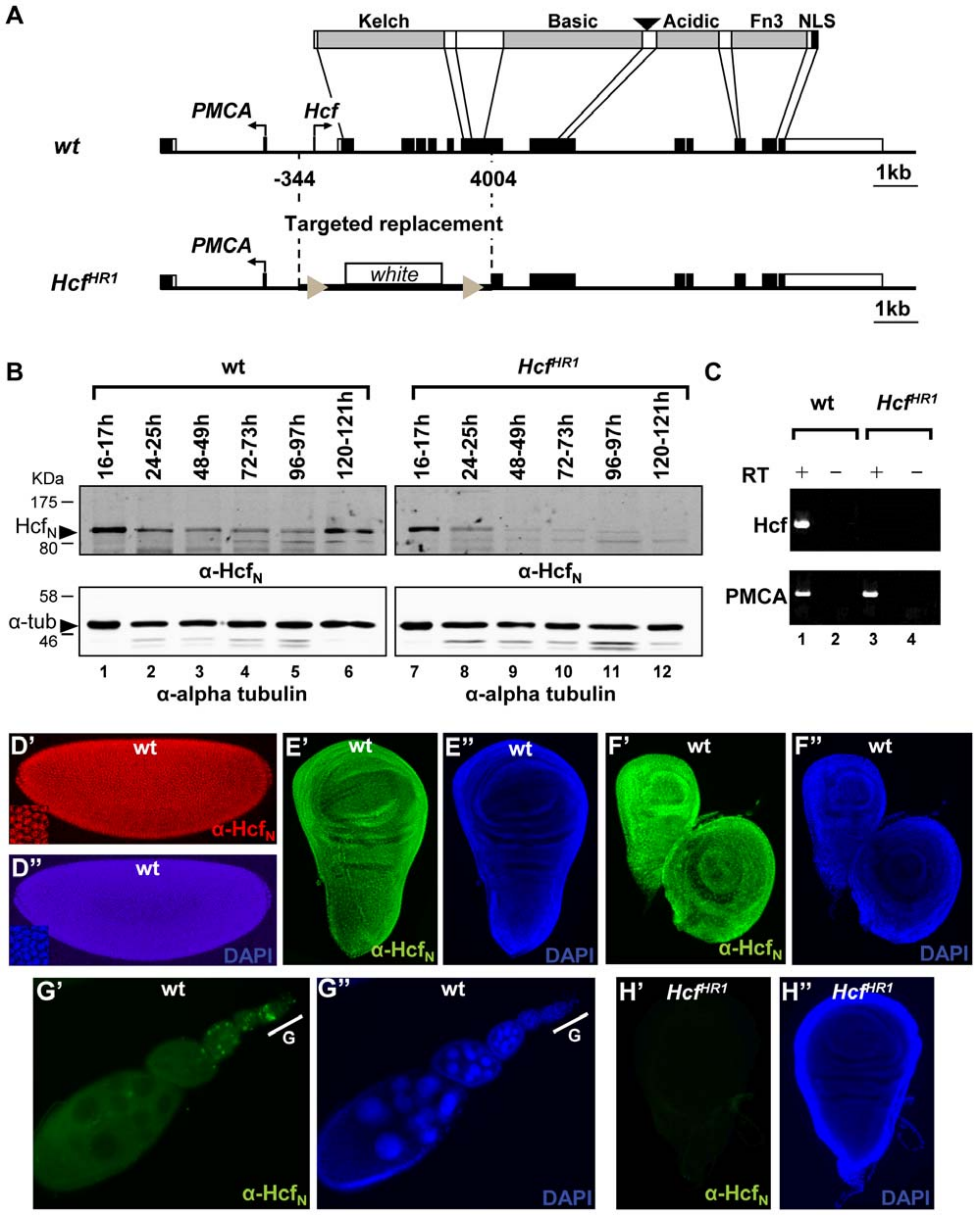
Structure and expression of wild-type *dHCF* gene and protein and mutant *dHCF^HR1^* allele. (A) Top. Illustration of the protein domains of *Drosophila melanogaster* dHCF. Fn3 - Fibronectin type 3 repeats, NLS - nuclear localization signal, arrowhead represents the taspase 1 proteolytic site. Middle. *dHCF* genomic region. Exons (white and black boxes), coding sequences (black boxes) and major transcription initiation sites (arrows) are shown. Bottom. *dHCF^HR1^* allele generated by homologous recombination. The boundaries of the genomic region replaced with the miniwhite gene are indicated by vertical dotted lines. Triangles represent loxP sites. (B) Protein extracts from wild-type and homozygous *dHCF^HR1^* embryos and larvae (indicated in hours after egg laying) were analyzed by immunoblotting with affinity purified anti-dHCF_N_ antibodies. (C) RT-PCR amplification of *dHCF* and PMCA RNAs from total RNA of wild-type and homozygous *dHCF^HR1^* third instar larvae. (D-H) Immunofluorescence analysis of (D) wild-type syncytial embryo, (E) wild-type third instar larva wing disc, (F) wild-type third instar larvae leg and haltere discs and (G) wild-type ovarioles, G – germarium (H) homozygous *dHCF^HR1^* third instar larva wing disc. Panels D'-H' show immunostaining with anti-dHCF_N_ antibodies and panels D''-H'' show DAPI staining of DNA. The inserts in D' and D'' show a magnified view of the image.

To study fly dHCF function, we undertook a multifaceted investigation of the *Drosophila dHCF* gene involving analyses of (i) *dHCF* expression, (ii) *dHCF* genetic disruption, and (iii) genetic *dHCF* interaction with known epigenetic regulators. The structures of the *dHCF* gene and encoded protein are shown in Figure 1A.

### *Drosophila dHCF* is broadly expressed throughout of development

Figure 1B shows an immunoblot analysis of the dHCF_N_ subunit at different embryo (lane 1) and larval (lanes 2-6) stages of wild-type flies. The dHCF_N_ and dHCF_C_ subunits (Fig. S1) were present at all stages, including adult (data not shown). Furthermore, immunostaining of embryos (Fig. 1D), imaginal discs (Fig. 1E and F) and ovaries (Fig. 1G) also revealed broad *dHCF* expression, with the dHCF protein localizing in the nucleus (see Fig. 1D insert for an example). The robust specificity of the affinity purified dHCF_N_ antibody for dHCF protein in immunofluorescence is shown in Figure S2. The broad pattern of *dHCF* expression suggests that the dHCF protein can have important roles throughout development. Pre-syncytial embryos and developing egg chambers (Fig. 1G) contain extensive levels of dHCF, which suggests that the protein and/or mRNA are maternally contributed to the embryo.

### Genetic disruption of *dHCF* by homologous recombination

The *Drosophila dHCF* gene is located on the highly heterochromatic and relatively poorly studied chromosome 4. Because there were no described *dHCF*-mutant alleles, we used ends-out homologous recombination [22] to generate the *dHCF^HR1^* knock-out allele (see Fig. S3), in which *dHCF* promoter sequences and exons 1 through 7 are replaced with the *mini-white* gene (Fig. 1A, bottom). Precise replacement was verified by PCR and sequence analysis (data not shown) and Southern blot analyses (Fig. S3C). Consistent with disruption of the *dHCF* gene, transcription of dHCF mRNA was not detected in homozygous *dHCF^HR1^* third-instar larvae, whereas the neighboring PMCA gene was apparently unaffected (Fig. 1C, compare lane 3 with lane 1). Suggesting maternal contribution of the dHCF protein or mRNA, analysis of dHCF subunit levels (Fig. 1B, lanes 7–12 and Fig. S1) in homozygous *dHCF^HR1^* offspring revealed a gradual loss of both dHCF subunits over the course of embryogenesis and larval development. Consistent with this extinction, larval imaginal discs stained negatively with dHCF antibodies in immunofluorescence assays (Fig. 1H). We used the *dHCF^HR1^* knock-out allele for the remainder of this study by generating *dHCF^HR1^* homozygous mutant individuals from *dHCF^HR1^/P{ActGFP}unc-13^GJ^* or *dHCF^HR1^/ciD* parents. Because *dHCF^HR1^* flies were extensively backcrossed onto *Df(1)w67c23, y^1^* flies, we used the latter as wild-type controls which are referred to as such.

### Loss of *dHCF* function results in lethality and sterility

Homozygous *dHCF^HR1^* animals survived until the pupal stage, where they exhibited an approximately 50% lethality, indicating that maternally derived dHCF reservoirs became limiting during pupal development. Of those surviving to adulthood, 30% of males and 100% of females were sterile.

**Figure 2:**
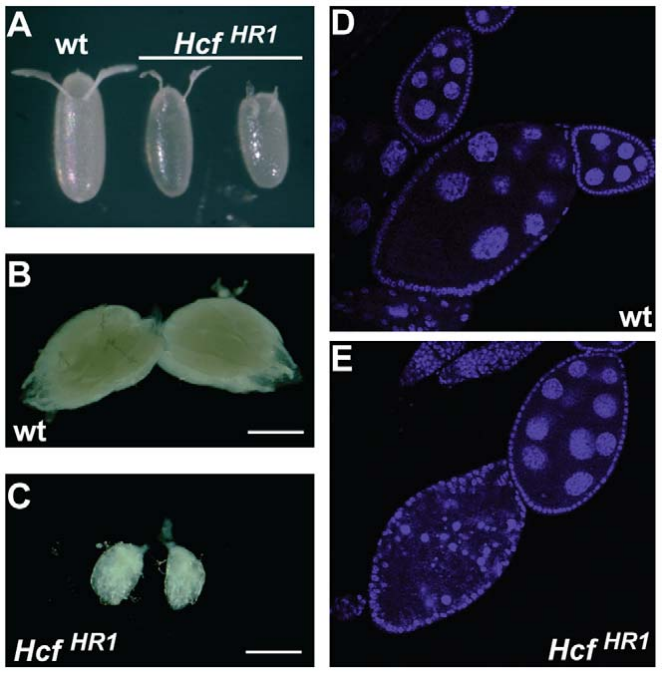
*dHCF* is essential for proper oogenesis. (A) Eggs laid by wild-type and homozygous *dHCF^HR1^* females. (B–C) Ovaries dissected from 4 day old (B) wild-type and (C) homozygous *dHCF^HR1^* female flies. (D–E) DAPI staining of fixed egg chambers from ovaries of 4 day old (D) wild-type and (E) homozygous *dHCF^HR1^* female flies.

Homozygous *dHCF^HR1^* females, while sterile, did lay some eggs, which were often fragile, smaller, and possessed misshapen anterior termini as well as dorsal appendages, as illustrated in Figure 2A. Consistent with this female sterility phenotype, the ovaries of homozygous *dHCF^HR1^* females were generally smaller and underdeveloped. The expressivity of this phenotype was variable and Figure 2 B and C illustrate an extreme example of this defect. We stained these ovaries with DAPI to examine the egg chambers in more detail and observed the punctuated pattern indicative of egg chamber degeneration around stage 8 of oogenesis, as illustrated in Figure 2D and E, suggesting that dHCF is essential for proper oogenesis.

### *dHCF* disruption leads to decreased size

**Figure 3:**
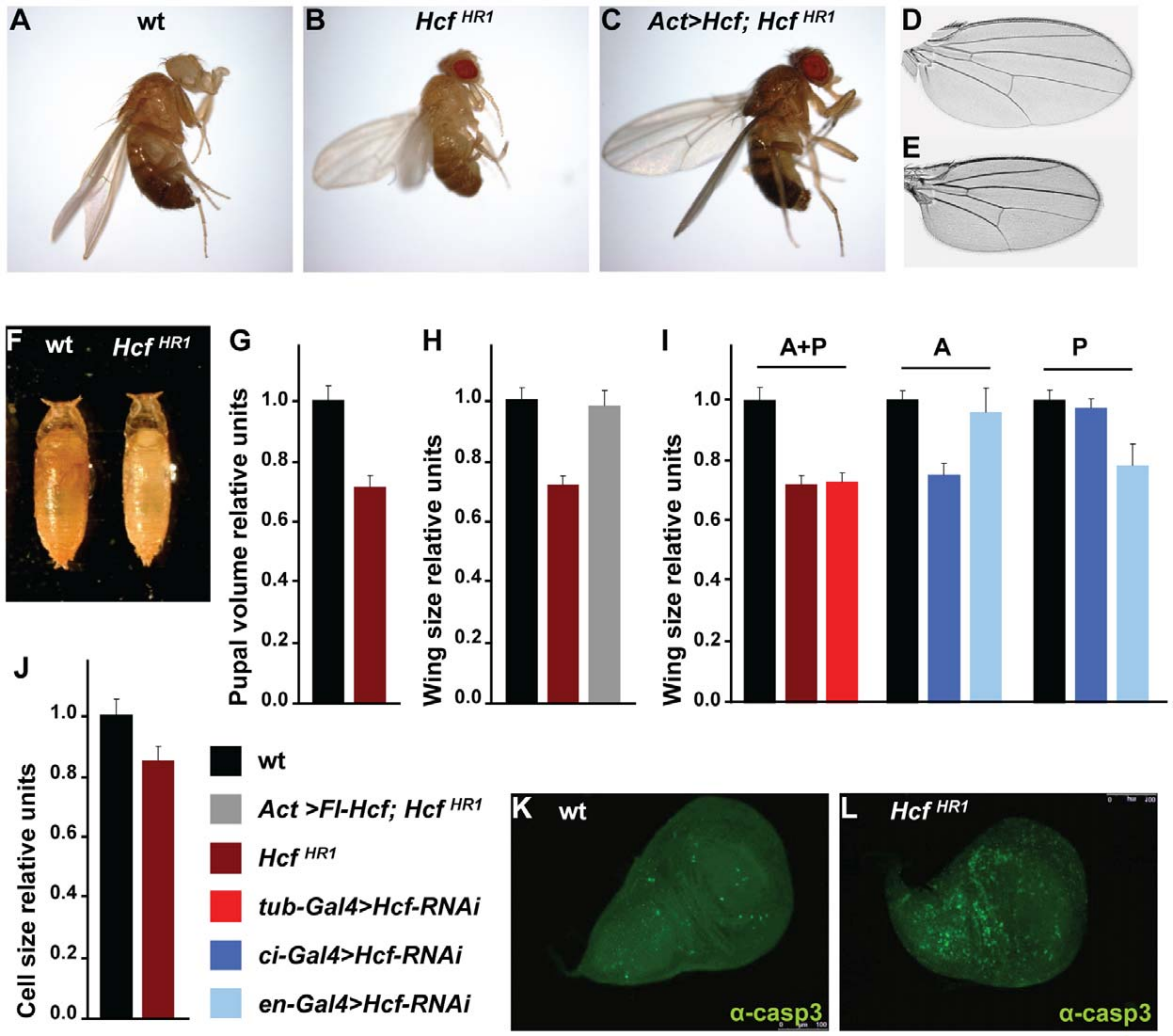
*dHCF* disruption results in a size reduction due to reduced cell size and increased apoptosis. (A–C) Pictures of (A) wild-type, (B) homozygous *dHCF^HR1^* and (C) rescue (*Act-GAL4/+; UAS-Fl-dHCF/+; dHCF^HR1^*) male flies grown at 18°C. (D–E) Pictures of wings from (D) wild-type and (E) homozygous *dHCF^HR1^* males. (F) Picture of (left) wild-type and (right) homozygous *dHCF^HR1^* male pupae. (G) Quantitation of pupal volume of wild-type and homozygous *dHCF^HR1^* male pupae in relative units. (H) Quantitation of total wing surface of wild-type, homozygous *dHCF^HR1^*, and rescue males grown at 18°C in relative units. (I) Quantitation of total wing surface (A+P), wing anterior compartment surface (A) and wing posterior compartment surface (P) of *UAS-dHCF-RNAi* flies carrying either the *tub-GAL4*, *ci-GAL4* or the *en-GAL4* drivers in relative units. (J) Quantitation of wing-cell size of wild-type and homozygous *dHCF^HR1^* males in relative units. (K–L) Immunofluorescence analysis of wing discs of third instar (K) wild-type and (L) homozygous *dHCF^HR1^* larvae using antibodies to activated caspase 3.

In addition to lethality and sterility, we also observed that homozygous *dHCF^HR1^* pupae and adults were consistently smaller than wild-type. Figure 3 shows side-by-side images of wild-type (Fig. 3A) and homozygous *dHCF^HR1^* adult males (Fig. 3B) and, similarly, wings (Fig. 3D and E) and male pupae (Fig. 3F). To quantify the size differences we compared pupal volume (Fig. 3G) and wing size (Fig. 3H). *dHCF^HR1^* homozygous pupae and adult wings were on average 30% and 28% smaller than wild-type, respectively.

We also analyzed the small-size wing phenotype using *UAS-dHCF-RNAi* transgenic flies [23]. When *dHCF* was broadly down-regulated in *tub-GAL4>UAS-dHCF-RNAi* flies, the *dHCF^HR1^* wing-size defect was fully phenocopied (Fig. 3I, left), as were other *dHCF^HR1^* phenotypes described below (e.g., loss of humeral bristles, extra wing vein) that were not quantified. We targeted *dHCF* knockdown to the posterior wing compartment in *en-GAL4>UAS-dHCF-RNAi* flies and to the anterior wing compartment in *ci-GAL4>UAS-dHCF-RNAi* flies, and measured the size of each wing compartment separately. As shown in Figure 3I (middle and right), we observed a reduction in size of the corresponding compartment with little effect on the other.

To demonstrate the specificity of these growth phenotypes on the loss of *dHCF* function, we generated and characterized wild-type and *dHCF^HR1^* flies with a full-length *dHCF* transgene (*UAS-Fl-dHCF*) under the control of either actin-GAL4 or tubulin-GAL4. We found that *Act-GAL4>UAS-Fl-dHCF; dHCF^HR1^* flies grown at 18°C were of normal body (Fig. 3C) and wing (Fig. 3H) size. This 18°C phenotypic rescue suggests that the size defect observed in homozygous *dHCF^HR1^* flies occurs as a consequence of loss of *dHCF* function. Enhanced expression of the *dHCF* transgene in *Act-GAL4>UAS-Fl-dHCF* or *Act-GAL4>UAS-Fl-dHCF; dHCF^HR1^* animals at 25°C resulted in male lethality and developmental abnormalities, including unextended or misshapen wings and absence of movement and oviposition in females. Additionally, overexpression of the *dHCF* transgene in *tub-GAL4>UAS-Fl-dHCF* or *tub-GAL4>UAS-Fl-dHCF; dHCF^HR1^* animals at either 18°C or 25°C resulted in a developmental delay and larval lethality, suggesting that ubiquitous *dHCF* over-expression can be disruptive to fly development (data not shown).

### *dHCF^HR1^* wings display reduced cell size and corresponding imaginal discs display increased apoptosis

The wing-size reduction of homozygous *dHCF^HR1^* flies could result from a reduction in cell size and/or in cell number. To determine the cause, we used the density of trichomes as a measure of wing-cell size in wild-type and *dHCF^HR1^* mutant wings. In the wings of homozygous *dHCF^HR1^* flies the trichome density increased by 18% compared to wild-type, indicating that the *dHCF^HR1^* mutant wing cells themselves are approximately 15% smaller than wild-type as shown in Figure 3J. This change in cell size does not account for the overall 30% decrease in mutant wing size, suggesting that the cell number is also affected.

We considered two possible explanations for a decrease in cell number in *dHCF^HR1^* wings: a reduction in cell proliferation or an increase in programmed cell death or apoptosis. To identify potential defects in cell proliferation, we performed FACS analysis on dissociated and propidium iodide-stained cells from wing discs of wild-type and homozygous *dHCF^HR1^* larvae. No differences were observed (data not shown). In addition, *in situ* measurement of S-phase cells by BrdU incorporation and M-phase cells by histone H3 phosphoserine 10 immunolabeling did not reveal significant changes between wild-type and homozygous *dHCF^HR1^* larvae (data not shown). In contrast, we did observe increased levels of apoptosis as indicated by an increase in the number of cells staining positively for activated caspase 3 as shown in Figure 3K and L. Acridine orange incorporation in live wing discs yielded similar results (data not shown). These results suggest that, in fly-wing development, the absence of dHCF protein does not significantly affect cell proliferation but does lead to increased apoptosis, which could contribute to the reduced size of *dHCF^HR1^* wings.

### *dHCF*-loss-of-function flies display TrxG-like and other developmental phenotypes

**Figure 4:**
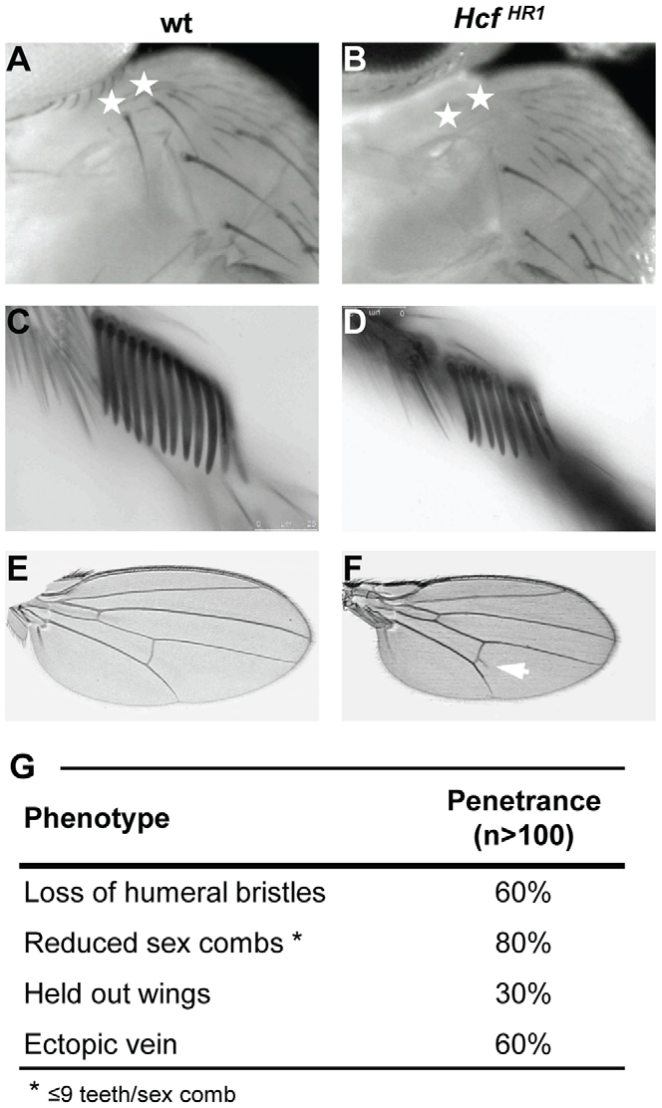
*dHCF^HR1^* flies display TrxG-like and other developmental phenotypes. (A–B) Humeral region of (A) wild-type and (B) homozygous *dHCF^HR1^* males. White stars indicate the position of humeral bristles, which are missing in homozygous *dHCF^HR1^* flies. (C–D) Examples of sex combs from (C) wild-type and (D) homozygous *dHCF^HR1^* males, carrying 12 and 8 teeth, respectively. (E–F) Wings of (E) wild-type and (F) homozygous *dHCF^HR1^* males. The arrowhead marks an ectopic vein starting from the posterior crossvein in *dHCF^HR1^* homozygous flies. (G) Observed penetrance of TrxG-related phenotypes in homozygous *dHCF^HR1^* flies compared to wild-type. Over 100 separate flies were included in the quantitation.

In addition to viability, sterility and size defects, homozygous *dHCF^HR1^* flies exhibited a series of phenotypes that are hallmarks for loss of TrxG function [24], [25], [26] including the homeotic phenotypes loss of humeral bristles (Fig. 4A and B), and sex-comb-tooth reduction, and the general developmental phenotype held-out wings in the resting position. The sex-comb-tooth reduction was from an average of 11.1 teeth in wild-type males to an average of 8.9 teeth in homozygous *dHCF^HR1^* males (Fig. 4C and D). As shown in Figure 4G, the penetrance of these phenotypes was incomplete. These results suggest that *dHCF* possesses TrxG-like properties.

In addition to the hallmark TrxG phenotypes, about 60% of homozygous *dHCF^HR1^* flies possessed an ectopic vein initiating from the posterior cross-vein (Fig. 4E–G). Ectopic veins initiating from the posterior cross-vein have also been observed in *SNR1* [27] and *corto* [28] mutants. *SNR1* encodes a component of the TrxG BRM complex and *corto* is a member of the ETP group of genes, further substantiating the hypothesis that the *dHCF* gene shares TrxG properties.

Importantly, the loss of humeral bristles and presence of held-out wings and ectopic veins, also present in *dHCF* flies grown at 18°C, were rescued by the *dHCF* transgene in *Act-GAL4>UAS-Fl-dHCF; dHCF^HR1^* flies grown at 18°C (data not shown). The rescue of the sex-comb tooth reduction phenotype was not examined. These results indicate that the pleiotropic TrxG-like and developmental phenotypes observed in *dHCF^HR1^* flies result from the loss of *dHCF* function.

### The *dHCF^HR1^* allele interacts with mutant alleles of the *brm* and *mor* TrxG genes

**Figure 5:**
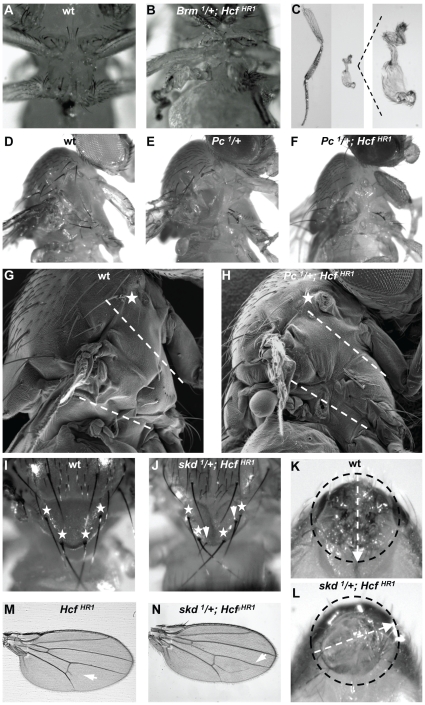
Genetic interaction between *dHCF* and TrxG and PcG genes. (A–C) Genetic interaction between *dHCF* and *brm*. Ventral view of the thorax of (A) wild-type and (B) *brm^1^/+; dHCF^HR1^* flies; (C) (left panel) metathoracic leg of the fly in (A) and (middle and right panel) metathoracic leg of the fly in (B). (D–H) Genetic interaction between *dHCF* and *Pc*. (D–F) Lateral thoracic view of (D) wild-type, (E) *Pc^1^/+* and (F) *Pc^1^/+; dHCF^HR1^* females. (G–H) Lateral SEM view of (G) wild-type and (H) *Pc^1^/+; dHCF^HR1^* females. The star indicates the first thoracic spiracle and the dashed lines frame the T2 thoracic segment. (I–N) Genetic interaction between *dHCF* and *skd*. (I–J) Scutellum of (I) wild-type and (J) *skd^1^/+;dHCF^HR1^* flies. The stars and arrowheads mark the position of scutellar and ectopic bristles, respectively. (K–L) Genitalia of (K) wild-type and (L) *skd^1^/+; dHCF^HR1^* males; extent of rotation is indicated by the arrow. (M–N) Wings of (M) *dHCF^HR1^* and (N) *skd^1^/+; dHCF^HR1^* males. The arrowheads mark ectopic veins starting from the posterior crossvein.

Having observed TrxG phenotypes in homozygous *dHCF^HR1^* flies, we asked whether the *dHCF^HR1^* allele might display interactions with mutant TrxG alleles. We generated flies homozygous for *dHCF^HR1^* and heterozygous for mutant alleles of four TrxG genes: *brm* and *mor*, which encode components of the SWI/SNF BRM complex, and *trx* and *Ash1*, which encode histone H3 methyltransferases (reviewed in [2]). Although we did not observe modification of TrxG homeotic phenotypes (data not shown), *brm^1^/+; dHCF^HR1^*, *brm^2^/+; dHCF^HR1^* and *mor^1^/+; dHCF^HR1^* flies often presented grossly misshapen metathoracic and, to a lesser extent, mesothoracic legs (see Figure 5A–C and Table 1). In addition, the leg position was shifted to a more dorsal position. Importantly, this phenotype was not observed in either one of *dHCF^HR1^*, *brm^1^*/+, *brm^2^*/+, or *mor^1^*/+ flies (Table 1). This result shows that the *dHCF* gene not only displays TrxG phenotypes when mutated but that it also interacts with TrxG genes encoding for the SWI/SNF nucleosome remodeling complex BRM.

**Table 1:** Genetic interaction between *dHCF* and TrxG, PcG and ETP genes.

Phenotype	Genotype	Penetrance	n
Leg defects (1)	*dHCF^HR1^*	0%	50
	*brm^1^ / +*	0%	50
	*brm^1^ / +; dHCF^HR1^*	20%	50
	*brm^20^ / +*	0%	50
	*brm^20^ / +; dHCF^HR1^*	10%	50
	*mor^1^ / +*	0%	50
	*mor^1^ / +; dHCF^HR1^*	10%	50
T2 to T1 transformation (2)	*dHCF^HR1^*	0%	64
	*Pc^1^ / +*	3%	72
	*Pc^1^ / +; dHCF^HR1^*	60%	65
	*Pc^3^ / +*	3%	68
	*Pc^3^ / +; dHCF^HR1^*	40%	52
Genitalia rotation defects (2)	*dHCF^HR1^ / dHCF^HR1^*	0%	100
	*Pc^1^ / +*	0%	100
	*Pc^1^ / +; dHCF^ HR1^*	3%	100
	*Pc^3^ / +*	0%	100
	*Pc^3^ / +; dHCF^ HR1^*	4%	104
	*Asx^XF23^ / +*	0%	100
	*Asx^XF23^ / +; dHCF^ HR1^*	2%	92
	*skd^1^*	0%	100
	*skd^1^ / +; dHCF^ HR1^*	30%	50
Ectopic vein (2)	*dHCF^ HR1^*	50%	64
	*Pc^1^ / +*	0%	72
	*Pc^1^ / +; dHCF^ HR1^*	5%	65
	*Pc^3^ / +*	0%	68
	*Pc^3^ / +; dHCF^ HR1^*	0%	52
Extra scutellar bristles (2)	*dHCF^ HR1^*	0%	50
	*skd^1^ / +*	0%	50
	*skd^1^ / +; dHCF^ HR1^*	30%	50

(1) This phenotype was quantified at 18°C and both eclosed and pharate adults were included. An observed increased mortality at the pharate state of *dHCF^HR1^* mutant combinations was likely due to leg defects.

(2) *dHCF^HR1^* mutant combinations displayed lethality phenotypes similar to *dHCF^HR1^* mutants.

### The *dHCF^HR1^* allele interacts with mutant PcG alleles

Because TrxG mutants are frequently defined by their ability to suppress PcG phenotypes, we examined whether the *dHCF^HR1^* mutation could suppress mutant phenotypes of the PcG founder gene, *Pc*. *Pc* mutants can display a variety of homeotic transformations including the appearance of sex combs in meta and mesothoracic legs in males, and, mostly in females, transformation of antenna-to-leg [29], abdominal segment A4 to A5 transformation, and reduction of the sternopleural region, which reflects a mesothoracic segment (T2) to prothoracic segment (T1) transformation [30] (see Figure 5D and E). Neither *Pc^1^/+; dHCF^HR1^* nor *Pc^3^/+; dHCF^HR1^* flies showed suppression or enhancement of extra sex combs or transformation of antenna to leg compared to *Pc^1^/+* and *Pc^3^/+* respectively (data not shown). Contrary to expectation, for sternopleural transformation, the *dHCF^HR1^* allele did not suppress the *Pc* phenotypes but instead the phenotype was observed at a much higher frequency in *Pc^1^/+; dHCF^HR1^* and *Pc^3^/+; dHCF^HR1^* flies than in either *Pc^1^/+* and *Pc^3^/+* flies, as quantified in Table 1. Figure 5 shows lateral views of sternopleurae of a wild-type fly (Fig. 5D) as well as representative examples of *Pc^1^*/+ (Fig. 5E) and *Pc^1^/+; dHCF^HR1^* (Fig. 5F) flies displaying a sternopleural transformation. To illustrate the T2 to T1 sternopleural transformation more clearly, we prepared scanning electron microscopy (SEM) high resolution images of wild-type and transformed *Pc^1^/+; dHCF^HR1^* sternopleurae, are shown in Figure 5G and H. These images reveal the reduction in size of the sternopleura (see region between broken lines). In addition, these *Pc^1^/+; dHCF^HR1^* flies also displayed defects in the first thoracic spiracle, which is a phenotype characteristic of double *Pc* and *Antp* (*Antennapedia*) mutants [31] (compare the first thoracic spiracle marked by the star in wild-type Fig. 5G and mutant Fig. 5H flies). Thus, the *dHCF^HR1^* allele, while displaying TrxG phenotypes, can unexpectedly also enhance rather than suppress a PcG phenotype. Thus, the *dHCF* gene displays both TrxG and PcG characteristics, a phenotype shared with the ETP group of genes.

We complemented the aforementioned study with an analysis of the effects of PcG mutants on *dHCF^HR1^* phenotypes. For this analysis, we selected mutants of subunits of the PcG complexes PRC1 (*Pc* alleles *Pc^1^* and *Pc^3^*) and PRC2 (*Enhancer of zeste* allele *E(z)^ su301^*). These mutations in the heterozygous state with *dHCF^HR1^* did not modify the penetrance and/or expressivity of the *dHCF^HR1^* adult size or humeral bristle phenotypes. The *Pc^1^* and *Pc^3^* mutants also did not modify the penetrance and/or expressivity of the sex comb size phenotype (data not shown). In contrast, as shown in Table 1, the *Pc^1^* and *Pc^3^* alleles suppressed the extra vein phenotype whereas *E(z)^su301^* had no effect. We also noted that approximately 3 to 4% of *dHCF^HR1^* males carrying the *Pc^1^* or *Pc^3^* alleles had defects in genitalia rotation (Table 1). In summary, there is a complex interaction with PcG mutations, in particular *Pc* itself.

### The *dHCF^HR1^* allele interacts with mutant alleles of the *skuld* ETP gene

We also examined the interaction between the *dHCF^HR1^* allele and mutant alleles of the ETP group genes *skuld* (*skd*), *Trithorax like* (*Trl*) and *Additional sex combs* (*Asx*). The *skd* gene product is a component of an accessory subcomplex of the Mediator [32] and the *Trl* gene product is the GAGA DNA-binding transcription factor [33], [34] and the *Asx* gene product interacts with the histone deubiquitinase Calyspo to form the PcG complex PR-DUB. No interaction was observed between *dHCF* and *Trl* while a small percentage of *Asx^XF23^*/+; *dHCF^HR1^* flies had genitalia rotation defects (see Table 1) As shown in Figure 5, *skd^1^*/+; *dHCF^HR1^* flies had ectopic bristles in the scutellum (Fig. 5I and J and Table 1) and genitalia rotation defects in males (Fig. 5K and L and Table 1), phenotypes that were not present in the single mutants. In addition, *skd^1^*/+; *dHCF^HR1^* flies showed increased expressivity of the wing-vein phenotype observed in homozygous *dHCF^HR1^* flies, as the extra-vein is considerably longer compared to *dHCF* single mutants (see Fig. 5M and N). These phenotypes suggest that the *dHCF* and *skd* / *Asx* genes, together, are important in suppressing vein and bristle formation as well as in genitalia development.

## Discussion

We have studied the role of dHCF in *Drosophila* development. By generating a targeted loss-of-function allele of the *dHCF* gene, we show that *dHCF* is an essential gene that when mutated has a pleiotropic phenotype, suggesting that dHCF is involved in a range of developmental pathways. The major phenotypes discerned include (i) female sterility, (ii) small size, and (iii) morphogenic alterations. These results suggest that dHCF plays a multifaceted role in the developmental regulation of gene expression. Consistent with a regulatory role, *dHCF* mutants display TrxG phenotypes and *dHCF* interacts genetically with PcG, TrxG and ETP epigenetic developmental regulators.

### HSV apparently targets a developmental regulator to control viral-gene transcription

PcG and TrxG proteins were first discovered via genetic studies in *Drosophila* and then via molecular studies were shown to belong to chromatin modifying complexes. In contrast, HCF-1 was first discovered via molecular studies of human viral gene expression, as a co-regulator of VP16-induced HSV immediate-early gene transcription [6], [7]. HCF-1 was subsequently shown to integrate human DNA-sequence-specific transcription factors and chromatin modifying complexes [8], [9], [10], [11], [12], [14], [15], [16] and these properties of HCF-1 are used to regulate HSV viral transcription [35]. The genetic analysis of *dHCF* in *Drosophila* clearly shows that HCF proteins possess broad developmental properties associated with epigenetic regulators. Thus, HSV apparently targets the developmental regulatory machinery in its interaction with HCF-1. Such regulation likely assists HSV in controlling the choice between lytic and latent states of infection.

### *dHCF* is an essential gene in *Drosophila*

Prior to this study, the only organism in which the *HCF* gene has been genetically disrupted, also by deletion, is in the worm *C. elegans*, where it is called *hcf-1* [36], [37]. The *hcf-1* mutant worms are viable at normal temperatures although they display fertility defects, cold-sensitive lethality, and increased longevity. The sterility and lethality phenotypes are more evident in the *Drosophila dHCF* mutant, perhaps owing to a more complex developmental program in this organism. In both worms [36], [37], [38], [39] and flies (this study), however, the *dHCF* mutant phenotypes are pleiotropic, suggesting that in both organisms HCF proteins are involved in a multitude of different developmental pathways. Together, these genetic results suggest that certain aspects of the developmental role of HCF proteins are shared, whereas others differ amongst species. Thus, as a developmental regulator, HCF proteins have probably assumed different gene regulatory roles during evolution.

### HCF proteins can contribute to growth using diverse mechanisms

Organism size is the product of cell growth, proliferation, and death. In human cells, HCF-1 has been implicated in cell proliferation and programmed cell death or apoptosis. For cell proliferation, it promotes the G1–S transition by association with and activation of the transcription factor E2F1 [8]. HCF-1 promotes apoptosis also via its interaction with E2F1 when the latter is disregulated [40]. In *Drosophila*, dHCF is also known to associate with E2F1 as well as the repressive E2F2 protein [8]. We have not detected, however, cell-cycle defects in imaginal discs lacking dHCF and loss of dHCF promotes apoptosis rather than inhibit apoptosis as in human cells [40]. Thus, HCF proteins indeed appear to possess different regulatory roles in humans and flies. As in *Drosophila* dE2F2 inhibits p53-independent radiation-induced apoptosis [41], perhaps dHCF inhibits apoptosis via dE2F2 as opposed to promote apoptosis in humans via E2F1. Whichever the case, these observations suggest that HCF proteins are implicated in different aspects of cell growth, proliferation and death in different species.

*Drosophila* dHCF apparently plays a role in cell-size determination as *dHCF* mutant flies show a reduction in cell size. Recently, Furrer et al. [42] have reported that the oncoprotein Myc and dHCF interact and that this interaction is important for the transcriptional activation and growth promoting functions of Myc. Indeed, Myc is a transcription factor that plays an important role in growth regulation [43]. The reduced cell size phenotype of *dHCF* mutant flies may be due to impaired function of Myc in the absence of dHCF proteins.

### *dHCF* interacts with TrxG, PcG, and ETP genes, positive and negative regulators of gene expression

The genetic analysis of *dHCF* loss of function presented here reveals pleiotropic collaboration with (i) the TrxG *brm* and *mor* genes for proper morphogenic regulation of leg development, (ii) the PcG *Pc* gene for proper specification of thoracic segment identity, and (iii) the ETP *skd* gene for bristle, vein and genitalia development. Consistent with these genetic interactions, dHCF can physically associate with the epigenetic TrxG protein Ash2 [21]. These findings in *Drosophila* are paralleled in human cells where HCF-1 associates with both TrxG – Set1 and MLL (human Trx) histone H3 lysine 4 methyltransferase complexes [14], [15] – and PcG – BAP-1 deubiquitinase (human Calypso) [3], [11], [44], [45] and YY1 (human Pho) [11] – -related factors. Thus, in both human and *Drosophila*, HCF proteins apparently interact with transcriptional regulators possessing roles in both activated and repressed states of gene expression.

The products of the identified *dHCF* collaborating genes exist in the cell in the form of multiprotein complexes – Brm and Mor are part of the BRM SWI/SNF complex [46], [47], Pc is a part of PRC1 [48], and skd [49] is a part of the Mediator – that possess transcriptional regulatory activities not previously associated with HCF proteins: the ATPase dependent chromatin remodeling activity of the BRM complex, the histone ubiquitination activity of PRC1, and the RNA polymerase II interaction activity of Mediator. These results portend more complex roles for HCF-1 in transcriptional regulation than currently appreciated and support the notion that HCF proteins are versatile integrators of gene regulatory information.

## Materials and Methods

### Fly stocks

Fly stocks were kept in standard corn/yeast media at 25°C unless otherwise indicated. The following alleles are described in flybase.org and available through the Bloomington Stock Center: *Df(1)w67c23, y^1^* (utilized as wild-type throughout this study), *ci^D^* and *P{ActGFP}unc-13^GJ^* (these two alleles were utilized throughout this study to maintain *dHCF^HR1^* stocks and distinguish homozygotes from heterozygotes), *tub-GAL4*, *en-GAL4*, *brm^1^*, *Act-GAL4*, *brm^20^*, *mor^1^*, *Pc^1^*, *Pc^3^*, *skd^1^*, *E(z)^ su301^*, *Asx^XF23^*. *UAS-dHCF-RNAi* transgenic flies (46998 and 46999) were obtained from the Vienna Drosophila RNAi Center. Stocks carrying the *UAS-dHCF RNAi* transgene on chromosome 2 and 3 were used in RNAi experiments. *ci-GAL4* flies were obtained from R.A. Holmgren's laboratory [50]. The generation of *dHCF^HR1^* and of *UAS-Fl-dHCF* alleles is described in Materials and Methods S1. For RNAi experiments, *UAS-dHCF-RNAi; UAS-dHCF-RNAi* flies were crossed with the GAL4 carrier stocks to generate *GAL4>UAS-dHCF-RNAi* flies. For the rescue experiments *UAS-Fl-dHCF; dHCF^HR1^ / ci^D^* flies were crossed with *Act-GAL4 / CyO; dHCF^HR1^ / ci^D^* flies to generate *Act-GAL4>UAS-Fl-dHCF ; dHCF^HR1^* flies.

### Protein extracts for immunoblotting (IB)

Protein extracts were prepared by cold homogenization of embryos or larvae in protein extraction buffer (50 mM TrisHCl pH 8, 150 mM NaCl, 1 mM EDTA, 4 mM EGTA, 0.1% SDS, 0.1% Triton X-100, 2X complete protease inhibitor cocktail (Roche)), followed by 10 min incubation on ice. Protein extracts were boiled for 5 min. in 1X Laemmli buffer, separated in Tris-Glycine acrylamide gels, transferred onto nitrocellulose Hybond membranes and probed with antibodies using the LI-COR system. Membranes were scanned with an Odyssey infrared imager (LI-COR).

### RNA extraction and Reverse Transcriptase (RT) PCR

RNA from third instar larvae was extracted using Trizol (Invitrogene) according to manufacturer specifications. cDNA was prepared by reverse transcription using the ImProm-II™ Reverse Transcription System (Promega) following manufacturer specifications. In addition, a negative control sample for genomic DNA amplification in the subsequent PCR reaction was prepared following the same protocol in the absence of reverse transcriptase. PCR amplification of cDNA or negative control sample was performed using the following primers for dHCF 5′-gatttatggtggaatgagcg-3′ and 5′-ctcgcacagacaagggatcag-3′ and for PMCA 5′-aaggcgtcacagtgtcagtactt-3′ and 5′-acatcttgaagagcctcccag-3′. PCR-amplified DNA fragments were resolved in agarose gels and visualized with ethidium bromide.

### Immunofluorescence (IF)

Embryos were dechorionated and fixed using standard methods [51]. Third instar wandering larvae were dissected in PBS to expose imaginal discs and fixed in (1∶3) 8% paraformaldehyde: Brower buffer (0.15 M PIPES, 3 mM Mg SO_4_ 1.5 mM EGTA, 1.5% NP-40 pH6.9) for 4h to overnight at 4°C. Ovaries were dissected from 3 day old well fed females in PBS and fixed in 3.7% formaldehyde in PBT for 20 min. Fixed tissue was washed in PBST (PBS-0.1% Tween 20) and rocked in blocking buffer (PBST with 1%BSA and 1% normal rabbit serum) for 1 h at RT to overnight at 4°C. Blocked tissue was incubated with primary antibodies in blocking buffer overnight at 4°C, washed in PBST, incubated with fluorescent secondary antibodies in blocking buffer for 1 hour at RT and washed in PBST followed by PBS before being mounted in VECTASHIELD® mounting medium with DAPI (Vectorlabs) to counterstain DNA. Images were taken with a microscope Leica DM6000B or a microscope Zeiss AXIO Vert 200 M with a Zeiss LSM 510 Meta confocal system.

### Scanning electron microscopy (SEM)

Three day old female flies were prepared for SEM by dehydration through increasing concentrations of ethanol at room temperature, and then dried with critical point evaporation of liquid CO2 (Balzers CPD 030). The samples were then attached with carbon cement to aluminum stubs and then metal coated with a 5–10 nm gold and palladium layer using high vacuum evaporation (Cressington Scientific). The samples were then imaged at 20 keV in a field emission SEM (FEI Company, XLF30-FEG).

### Viability assays

Embryos were collected and let hatch on fruit juice-agar plates and first instar larvae counted and transferred to fruit juice-agar plates with standard corn/yeast media to quantify larval viability or to standard corn/yeast media tubes to quantify pupal and adult viability. Viability of a given developmental phase was calculated as the ratio between the number the individuals exiting and the number of individuals entering that phase.

### Fertility assays

Two day old virgin females (n = 40) or males (n = 40) of the appropriate genotype were individually mated with two wild-type males or virgin females, respectively. After two days, eggs were counted daily for a three day period. Individuals were considered sterile when they laid, in total, less than 5% of the number of eggs laid by wild-type females mated with wild-type males.

### Wing, wing cell and pupal size quantification

Wings of male flies were dissected, mounted in Euparal and photographed in a microscope Leica DM6000B. Pictures taken with the 5X objective were used to determine the area using the Image J software. The fourth vein was used as border between posterior and anterior compartments. Pictures of a specific area in the wing were taken with the 40X objective and used to determine manually using Image J the trichome number per surface unit or cell density. The relative cell size was calculated as the reverse of the cell density. Pupal volume was determined as in Layalle et al. using the formula 4/3π(L/2)(l/2)^2^ (L, length; l, diameter) [52].

### Antibodies

The following primary antibodies were used: rabbit anti-dHCF_N_ (affinity purified, IB 1∶500, IF 1∶50) [19], mouse anti-α-tubulin (Clone B-5-1-2, Sigma, IB 1∶5000), mouse anti-activated-caspase 3 (Cell Signaling, IF 1∶50), Alexa 680- and IRDye 800-conjugated secondary antibodies (Molecular Probes and Rockland Immunochemicals, IB 1∶10,000–20,000), Alexa 488- and Alexa 543-conjugated secondary antibodies (Molecular Probes, IF 1∶500).

## Supporting Information

Figure S1 **Analysis of the dHCF_C_ subunit during development in wild-type and** ***dHCF^HR1^*** **mutants.** Protein extracts from wild-type and homozygous *dHCF^HR1^* embryos and larvae (indicated in hours after egg laying) were analyzed by immunoblotting with anti-dHCF_C_ antibodies. The same blots were incubated with α-tubulin antibodies to control for protein loading. Star, non-specific band of unknown origin.
(PDF)

Figure S2 **Specificity of RNAi inactivation of** ***dHCF*** **and of the dHCF_N_ antibody for immunofluorescence.** Wing imaginal disc of *ci-GAL4/ UAS-dHCF-RNAi; UAS-dHCF-RNAi / UAS-GFP* third instar wandering larvae. (A) Immunostaining with dHCF_N_ antibodies. (B) GFP fluorescence. (C) DAPI staining. Note specific loss of dHCF_N_ immunofluorescence in GFP-positive cells.
(PDF)

Figure S3 ***dHCF*** **gene deletion by ends out homologous recombination.** (A) Schematic drawing illustrating the *dHCF*-gene structure. Represented are exons (white and black boxes), coding sequence (black boxes) and the main transcription initiation site (arrow). Restriction sites and probes used in the Southern blot analysis shown in (C) are shown under the line. Bx-BstX I, S-Sal I, N-Nde I, B-BamH I. (B) Targeting vector and structure of the *dHCF* genomic region after homologous recombination. Numbers indicate the position of the sequence with respect to the *dHCF* transcription-initiation site. Dotted boxes represent regions of identity between the targeting vector and *dHCF* gene locus. (C) Southern blot analysis of *dHCF^HR1^* recombinant flies: genomic DNA from wild-type and heterozygous *dHCF^HR1^/+* males was digested with the indicated enzymes and detected by Southern blotting using the indicated probes.
(PDF)

Materials and Methods S1 Supporting materials and methods.
(DOC)
