# Supplementary figures and images for: Drosophila melanogaster dHCF Interacts with both PcG and TrxG Epigenetic Regulators
Source: PLoS One. 2011 Dec 8;6(12):e27479. doi: 10.1371/journal.pone.0027479 (PMC3234250; doi:10.1371/journal.pone.0027479)

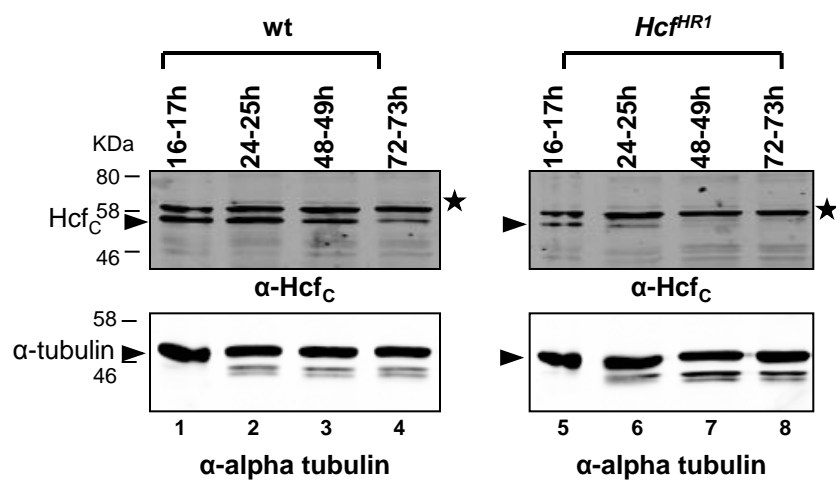

Supplement: Figure S1 — Analysis of the dHCFC subunit during development in wild-type and dHCFHR1 mutants. Protein extracts from wild-type and homozygous dHCFHR1 embryos and larvae (indicated in hours after egg laying) were analyzed by immunoblotting with anti-dHCFC antibodies. The same blots were incubated with α-tubulin antibodies to control for protein loading. Star, non-specific band of unknown origin. (PDF) [file pone.0027479.s001.pdf]

*ci-GAL4 / UAS-Hcf-RNAi; UAS-Hcf-RNAi / UAS-GFP*

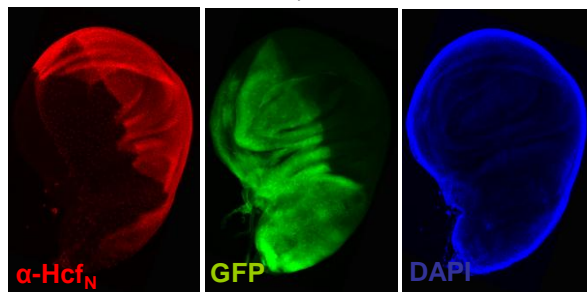

Figure S2

Supplement: Figure S2 — Specificity of RNAi inactivation of dHCF and of the dHCFN antibody for immunofluorescence. Wing imaginal disc of ci-GAL4/ UAS-dHCF-RNAi; UAS-dHCF-RNAi / UAS-GFP third instar wandering larvae. (A) Immunostaining with dHCFN antibodies. (B) GFP fluorescence. (C) DAPI staining. Note specific loss of dHCFN immunofluorescence in GFP-positive cells. (PDF) [file pone.0027479.s002.pdf]

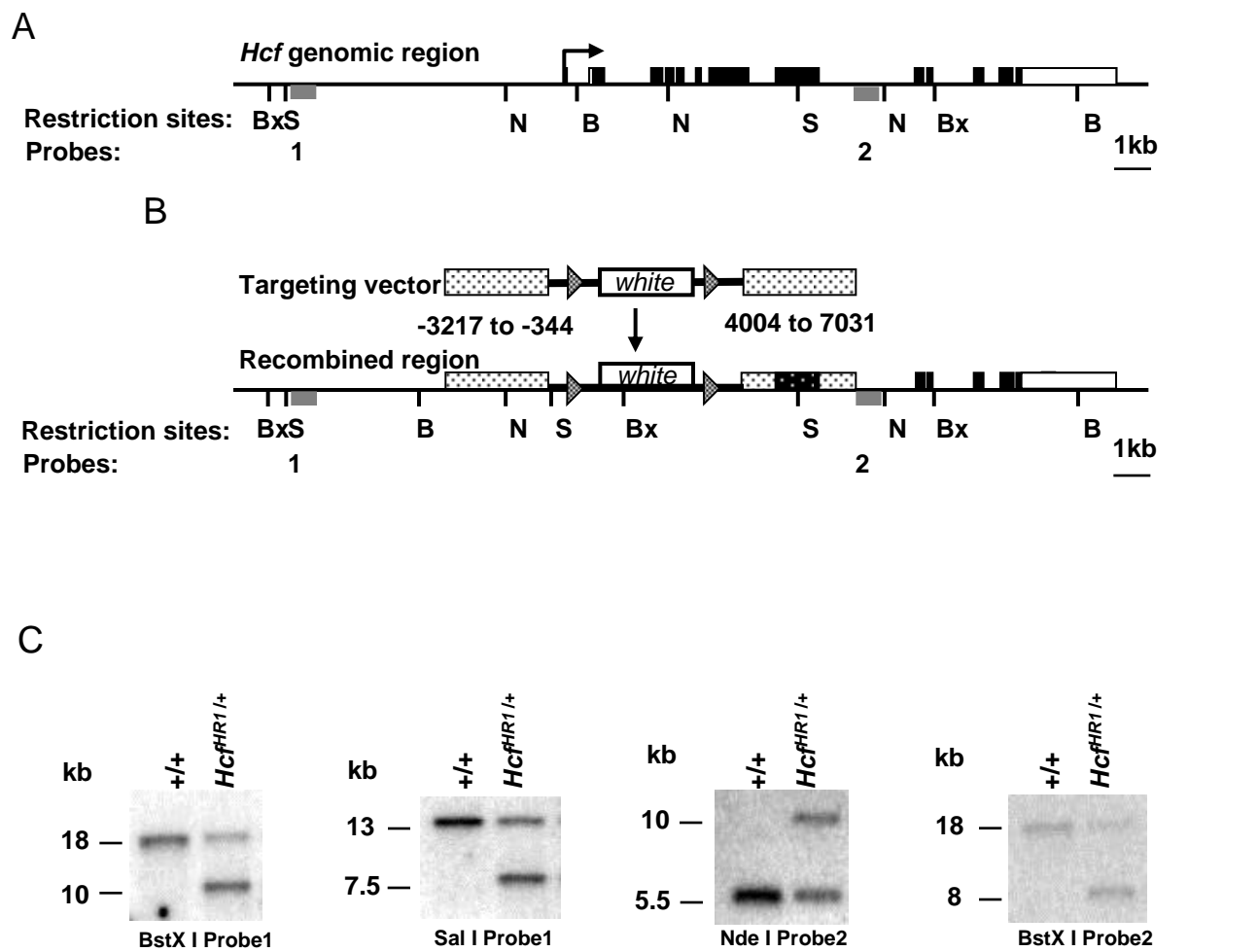

Figure S3

Supplement: Figure S3 — dHCF gene deletion by ends out homologous recombination. (A) Schematic drawing illustrating the dHCF-gene structure. Represented are exons (white and black boxes), coding sequence (black boxes) and the main transcription initiation site (arrow). Restriction sites and probes used in the Southern blot analysis shown in (C) are shown under the line. Bx-BstX I, S-Sal I, N-Nde I, B-BamH I. (B) Targeting vector and structure of the dHCF genomic region after homologous recombination. Numbers indicate the position of the sequence with respect to the dHCF transcription-initiation site. Dotted boxes represent regions of identity between the targeting vector and dHCF gene locus. (C) Southern blot analysis of dHCFHR1 recombinant flies: genomic DNA from wild-type and heterozygous dHCFHR1/+ males was digested with the indicated enzymes and detected by Southern blotting using the indicated probes. (PDF) [file pone.0027479.s003.pdf]
