# Supplementary material for: Drosophila melanogaster dHCF Interacts with both PcG and TrxG Epigenetic Regulators
Source: PLoS One. 2011 Dec 8;6(12):e27479. doi: 10.1371/journal.pone.0027479 (PMC3234250; doi:10.1371/journal.pone.0027479)
Supplement: Materials and Methods S1 — Supporting materials and methods. (DOC) [file pone.0027479.s004.doc]

**MATERIALS AND METHODS S1**

**Generation of *dHCF* mutants by ends out homologous recombination.** The injection construct, SR-PW4, was generated from the pW25 vector and contained the sequences of the 5’ and 3’ homology arms which spanned from position -3217 to -344 and from +4004 to +7031, respectively, respect to *dHCF*’s transcription initiation site. These sequences were amplified from genomic DNA by PCR using a mix of Taq and Pfu DNA polymerases and primers 5’-CGAACTGACCATGCCCGTC-3’ and 5’-TTCGAATTAATATTTGTGGTGCGA-3’ for the 5’ homology arm and 5’- TTACGCGTGGAGCTCTCGTGGACTGCG -3’ and 5’- TTACGCGTGGTTGTGGCAGAATGGCTG -3’ for the 3’ homology arm. The PCR product containing 5’ homology arm sequences was cloned into a pBluescript-TA vector (pBluescript was digested with EcoRV and incubated with Taq and dTTP to create T overhangs) and then subcloned into pW25 using Kpn I and Not I to generate SR-PW25-5’. The PCR product containing 3’ homology arm sequences was digested with Mlu I and cloned into an Asc I site of SR-PW25-5’ to generate the final donor construct SR-PW4. Injections were performed by Genetic Services, Inc. using *w^118^* embryos as host to generate donor flies *w^118^; SR-PW4* which carried the transgene in the second chromosome. Larvae from a *w^118^; SR-PW4* x *y^1^ w^*^; P{70FLP}11 P{70I-SceI}2B noc^Sco^/CyO, S^2^* cross were heat shocked for 90 min at 37°C on day 3 after egg laying. Female offspring were crossed to *w^1118^; P{70FLP}10* males, and offspring were screened for white^+^ eyes. Successful targeting events into the *dHCF* locus were verified by PCR and Southern analysis (see below). Two independent recombination events were named *dHCF^HR1^* and *dHCF^HR2^*. Partial recombination events that led to integration of the targeting sequences without excision of the genomic region were also recorded. *dHCF^HR1^/+* flies were extensively crossed to *Df(1)w67c23, y^1^* and then to *ci^D^/pho^1^* to obtain the stock *dHCF^HR1^/* *ci^D^*.

**Molecular characterization of targeted events:** PCR analysis of homologous recombination events was performed using a primer to the genomic sequence adjacent to the homology arm and a primer within the sequence of pW25: 5’-TTTACACCCGTTTCTCGTTGAA-3’ and 5’-GGGGCATGATAACTTCGTATAGC-3’ for the 5’ region and 5’-CTTGGCTGCAGGTCGACTCT-3’ and 5’-CGGGACAAAAGAGCCAAGC-3’ for the 3’ region. Southern analysis was performed with genomic DNA from heterozygous *dHCF^HR1^/+* male flies. DNA was digested with BstX I, Sal I or Nde I, separated by agarose gel electrophoresis and transferred to Hybond N+ membranes. Probes were derived from PCR products amplified from genomic DNA using the following primers 5’-GACCCAGCATATCCAATCTG-3’ and 5’-TTTGGGCAAATGTATAGAAACA-3’ for probe1 and 5’-TACATGGACCTTAGCTTGGCT-3’ and 5’-TGTGTACAAAACTTTGCTAGAAGC-3’ for probe2.

**Generation of *UAS-Fl-dHCF* transgenic flies:** *UAS-Fl-dHCF* transgenic flies were generated using the φC31 integrase transgenesis system described in Bishof et al. . Purified injection construct, pa-T7-Fl-dHCF-Flag, was injected into y w hsFlp; M{RFP.attP}ZH-86Fb; M{eGFP.vas-int.Dm}ZH-102D flies using standard procedures .

*dHCF* coding sequences used in the generation of pa-T7-Fl-dHCF-Flag were derived from the vector pACXT-dHCF as follows. dHCF C-terminal sequences were subcloned from pACXT-dHCF into pBluescript with Xho I to generate pB-dHCF-X. A double stranded oligonucleotide containing *dHCF* and Flag tag sequences (5’-GCGTGGACGCAACGGATTGCATGATGCTAGCGATTACAAGGATGACGACGATAAGTAGGTACCTACGCGT -3’) was cloned between the two Ale I sites of pB-dHCF-X to generate pB-dHCF-X2-Flag. T7-tagged dHCF_N_ subunit was PCR amplified from pACXT-dHCF using 5’- TTTTACTAGTATGGCTTCTAGGATGGCATCG -3’ and 5’- TAATACTAGTTAATCCAACTGCTCTATAATATCATCCAT -3’. The amplified product was cloned into pUASTattB using Spe I to generate pa-T7-dHCF_N_. The Flag-tagged dHCF_C_ subunit was then cloned from pB-dHCF-X2-Flag into pa-T7-dHCFN with EcoR I to generate pa-T7-Fl-dHCF-Flag.

**REFERENCES**

**Bischof, J., Maeda, R. K., Hediger, M., Karch, F. and Basler, K.** (2007). An optimized transgenesis system for Drosophila using germ-line-specific phiC31 integrases. *Proc Natl Acad Sci U S A* **104**, 3312-7.

**Mahajan, S. S., Johnson, K. M. and Wilson, A. C.** (2003). Molecular cloning of Drosophila HCF reveals proteolytic processing and self-association of the encoded protein. *J Cell Physiol* **194**, 117-26.

**Rong, Y. S., Titen, S. W., Xie, H. B., Golic, M. M., Bastiani, M., Bandyopadhyay, P., Olivera, B. M., Brodsky, M., Rubin, G. M. and Golic, K. G.** (2002). Targeted mutagenesis by homologous recombination in D. melanogaster. *Genes Dev* **16**, 1568-81.

**Sullivan, W., Ashburner, M. and Hawley, R.** (2000). *Drosophila* Protocols: Cold Spring Harbor Laboratory Press.
